# A pilot sentinel surveillance system to monitor treatment and treatment outcomes of chronic hepatitis B and C infections in clinical centres in three European countries, 2019

**DOI:** 10.2807/1560-7917.ES.2023.28.6.2200184

**Published:** 2023-02-09

**Authors:** Anthony Nardone, Lina Nerlander, Erika Duffell, Marta Valenciano, Maria Buti, Cristina Marcos-Fosch, Tatjana Nemeth-Blažić, Odette Popovici, Adriana Vince, Petruta Violeta Filip, Tajana Filipec, Mirjana Lana Kosanović Ličina, Boris Luksic, Diana Nonković, Corina Silvia Pop, Fabiana Radu, Irina Teodorescu, Adriana Violeta Topan

**Affiliations:** 1 Epiconcept, Paris, France; 2 European Centre for Disease Prevention and Control (ECDC), Stockholm, Sweden; 3 Hospital Universitario Valle Hebrón & CIBEREHD del Instituto Carlos III, Barcelona, Spain; 4 Croatian Institute of Public Health, Zagreb, Croatia; 5 National Institute of Public Health, Bucharest, Romania; 6 University Hospital for Infectious Diseases, Medical School University of Zagreb, Croatia; 7 University Emergency Hospital, Bucharest, Romania; 8 Clinical Hospital Merkur, School of Medicine, University of Zagreb, Zagreb, Croatia; 9 Andrija Stampar Teaching Institute of Public Health, Zagreb, Croatia; 10 Clinical Hospital Centre Split, School of Medicine University of Split, Split, Croatia; 11 Teaching Institute of Public Health Split and Dalmatia county, University Department of Health Studies, Split, Croatia; 12 Clinical Infectious Diseases Hospital, Iasi, Romania; 13 “Iuliu Hatieganu” University of Medicine and Pharmacy, Hospital of Infectious Diseases, Cluj-Napoca, Romania

**Keywords:** hepatitis B, hepatitis C, treatment, surveillance

## Abstract

**Background:**

The World Health Organization European Action Plan 2020 targets for the elimination of viral hepatitis are that > 75% of eligible individuals with chronic hepatitis B (HBV) or hepatitis C (HCV) are treated, of whom > 90% achieve viral suppression.

**Aim:**

To report the results from a pilot sentinel surveillance to monitor chronic HBV and HCV treatment uptake and outcomes in 2019.

**Methods:**

We undertook retrospective enhanced data collection on patients with a confirmed chronic HBV or HCV infection presenting at one of seven clinics in three countries (Croatia, Romania and Spain) for the first time between 1 January 2019 and 30 June 2019. Clinical records were reviewed from date of first attendance to 31 December 2019 and data on sociodemographics, clinical history, laboratory results, treatment and treatment outcomes were collected. Treatment eligibility, uptake and case outcome were assessed.

**Results:**

Of 229 individuals with chronic HBV infection, treatment status was reported for 203 (89%). Of the 80 individuals reported as eligible for treatment, 51% (41/80) were treated of whom 89% (33/37) had achieved viral suppression. Of 240 individuals with chronic HCV infection, treatment status was reported for 231 (96%). Of 231 eligible individuals, 77% (179/231) were treated, the majority of whom had received direct acting antivirals (99%, 174/176) and had achieved sustained virological response (98%, 165/169).

**Conclusion:**

Treatment targets for global elimination were missed for HBV but not for HCV. A wider European implementation of sentinel surveillance with a representative sample of sites could help monitor progress towards achieving hepatitis control targets.

**Key public health message**

**What did you want to address in this study?**

To eliminate viral hepatitis, the World Health Organization recommends achieving high levels of treatment (>75% of eligible individuals) and viral suppression (>90% of those treated). Yet limited data on treatment uptake and outcome are available. We conducted a pilot sentinel surveillance in seven clinical centres in three countries to monitor progress in achieving treatment targets Europe.

**What have we learnt from this study?**

In the seven sites participating in this pilot, in patients with chronic hepatitis C attending a clinic for the first time, treatment uptake (77%) and viral suppression (98%) met international targets. However, both targets were missed (51% and 89%, respectively) for patients attending for the first time with chronic hepatitis B.

**What are the implications of your findings for public health?**

The problems of low uptake of hepatitis B treatment need to be addressed. This pilot sentinel surveillance demonstrated that the data collected provided insights into treatment and treatment outcomes of patients diagnosed with hepatitis B or C not currently obtained by existing routine data collection, especially in providing data on the continuum of care in each country.

## Introduction

Chronic hepatitis B virus (HBV) and hepatitis C virus (HCV) infections can progress to liver cirrhosis and hepatocellular carcinoma (HCC) [1,2]. Both HBV and HCV are a major cause of morbidity and mortality and were responsible for an estimated 1.1 million deaths globally in 2019 [3]. It is estimated that in 2015 within the European Union (EU) and the European Economic Area (EEA) (including the United Kingdom at the time) there were ca 4.7 million people living with chronic HBV infection, 3.9 million with chronic HCV infection and around 64,000 deaths [4,5].

Treatment is one of the core components of the World Health Organization (WHO) strategy for the elimination of viral hepatitis by 2030 [6]. Targets outlined in the WHO European Action Plan for the elimination of hepatitis recommend 75% of eligible patients are treated, and 90% of those treated reach viral suppression [7]. The WHO Global Health Sector Strategy target stipulates that 80% of eligible patients should be treated by 2030 [8]. The introduction of direct acting antivirals (DAA) has provided a safe, effective and short treatment for HCV [9]. For HBV, longer term treatment with nucleos(t)ide analogues can suppress viral replication and reduce morbidity and mortality [10]. Hepatitis treatment provides an additional approach to prevent and control hepatitis, especially for HCV where no vaccine is currently available [11]. The introduction of DAA offers not only individual benefit but has made HCV treatment as prevention a viable and realistic option [12].

Information for focussed action is a key pillar of the WHO European Action Plan, and that requires national surveillance programmes to generate high-quality data on outbreaks, incidence and treatment and care [7]. Surveillance data, based on the notification of newly diagnosed acute and chronic cases of HBV and HCV, are collected by EU/EEA countries and collated by the European Centre for Disease Prevention and Control (ECDC) [13,14]. Surveillance data are often limited by under-reporting, lack of completeness of many variables [13,14], and, as they are often not longitudinal, information is unavailable on treatment and treatment outcomes. In addition to collecting the hepatitis surveillance data, the ECDC began to monitor the national progress of EU/EEA countries in reaching the elimination targets for HBV and HCV through the collection and collation of epidemiological data, information on prevention strategies as well as testing and treatment indicators for the continuum of care [15]. The first data collected in 2019 highlighted major gaps in national hepatitis surveillance data, particularly in relation to prevention, testing and treatment. Thus, there is a need to enhance and supplement the national surveillance systems with data from alternative surveillance schemes or other data sources.

The ECDC funded and supported a pilot sentinel surveillance project for the collection of a limited set of detailed, high quality clinical and laboratory data, including treatment outcome, from patients with chronic HBV and HCV infections attending clinics for care. The aim of the project was to pilot a European sentinel surveillance for viral hepatitis, with particular focus on monitoring progress towards WHO treatment targets, to enhance and supplement existing surveillance data in order to inform the prevention and control measures for these infections. We present data related to treatment and treatment outcomes of individuals with chronic HBV and HCV infections.

## Methods

### Study design

The pilot sentinel surveillance system was established in hospital clinics and employed a retrospective cohort design in which anonymous data were extracted from medical records of patients with a confirmed diagnosis of chronic HBV or HCV presenting for the first time at any of the participating clinics between 1 January 2019 and 30 June 2019. The study design and protocol were developed using information from a rapid literature review, a feasibility assessment, interviews with selected ECDC National Focal Points from EU/EEA countries and experts from the ECDC and the European Association for the Study of the Liver (EASL), and discussions with public health and clinical experts from the three pilot countries Croatia, Romania and Spain. Seven sentinel sites were purposively selected based in part on their interest in participating in this pilot. The site principal investigators provided contextual information on catchment areas, local clinical pathways and treatment policies (Table 1).

**Table 1:** Participating clinics by type of service, catchment population and reported chronic hepatitis B and chronic hepatitis C cases, Croatia, Romania, Spain, January–June, 2019

Country and clinic	Type of service		Catchment population		Chronic HBV infections reported		Chronic HCV infections reported	
	Medical speciality	Service level	Catchment area	Size catchment population	Number	%	Number	%
**Croatia**					**24**	**10**	**92**	**38**
Clinical Hospital Centre, Split	Infectious disease	Tertiary	Regional	455,000	9	NA	35	NA
Clinical Hospital Merkur, Zagreb	Gastroenterology/national liver transplant service	Tertiary	National	4,100,000	3	NA	10	NA
University Hospital for Infectious Diseases Dr Fran Mihaljević (UHID), Zagreb	Infectious disease	Tertiary	National	1,000,000	12	NA	48	NA
**Romania**					**150**	**66**	**95**	**40**
University Emergency Hospital, Bucharest	Gastroenterology	Tertiary	National	304,000	141	NA	47	NA
Clinical Infectious Diseases Hospital, Cluj-Napoca	Infectious disease	Tertiary	Regional	36,000	1	NA	2	NA
Clinical Infectious Diseases Hospital, Iasi	Infectious disease	Tertiary	Regional	104,000	8	NA	46	NA
**Spain**					**55**	**24**	**53**	**22**
Hospital Universitario Valle Hebrón, Barcelona	Hepatology	Tertiary	District	450,000	55	NA	53	NA
**Total**					**229**	**100**	**240**	**100**

HBV: hepatitis B virus; HVC: hepatitis C virus; NA: not applicable.

### Case definition

Sentinel sites reported all individuals who presented for the first time between 1 January and 30 June 2019 with an HBV and/or HCV diagnosis that conformed with the European Union (EU) 2018 case definitions [16]. Additional ECDC criteria for chronic HBV or HCV infection were modified and applied to chronic cases reported by sites [14,15]. Reported HBV chronic infections were confirmed if hepatitis B surface antigen (HBsAg), hepatitis B e antigen (HBeAg) or hepatitis B nucleic acid (HBV DNA) were detected and IgM hepatitis B core antibody (anti-HBc IgM) was negative or not reported [14]. Reported HCV chronic infections were confirmed if hepatitis C nucleic acid (HCV RNA) or hepatitis C core antigen (HCVc Ag) were detected and hepatitis C specific-antibody (anti-HCV) was positive [15]. To accommodate the high levels of missing data, individuals with ‘no report’ of either anti-HBc IgM or anti-HCV were still defined as having a chronic infection if reporting one or more of the other criteria.

### Treatment eligibility criteria

All individuals with chronic HCV were considered eligible for treatment as outlined in current European guidelines [17]. For HBV, treatment eligibility was reported by local collaborators and further assessed by employing the following treatment eligibility criteria recommended by the EASL for the management of HBV infection [18]: (i) reported diagnosis of cirrhosis; (ii) chronic hepatitis defined as either HBeAg-positive or negative and HBV-DNA > 2,000 IU/ml and/or alanine aminotransferase (ALT) > 40IU/ml and/or at least moderate fibrosis (F2 or greater); (iii) very high HBV-DNA (defined as > 20,000 IU/ml) and ALT, defined as twice upper limit normal (ULN; > 80IU/ml); (iv) HBeAg positive, normal ALT (< 40IU/ml), high HBV-DNA (> 2,000 IU/ml) and aged 30 years or older and/or a family history of HCC (although data were not collected on the last criterion); (v) other reasons including prevention of mother-to-child transmission or reactivation due to immunosuppression or chemotherapy.

### Data collection

Clinical records of new patients who presented at sentinel sites for the first time between 1 January 2019 and 30 June 2019 were reviewed from date of first attendance to 31 December 2019. Data extraction was undertaken by study collaborators based at the sentinel sites using standardised data extraction forms. Between March and August 2020, data were submitted through online entry to the study coordinators using the Voozanoo platform [19] and access was limited to study collaborators.

Anonymous data on patients were collected for the following domains: patient variables, laboratory variables, clinical history at presentation and treatment.

For patient variables, data on age, sex, country of birth and likely route of transmission were collected using the same specification required for submission of notifications of newly diagnosed cases of hepatitis B and C to the European Surveillance System managed by the ECDC [13,14]. Routes of transmission were recoded according to the infection. For HBV infections, non-occupational routes of transmission included household contact, injecting drug use and other routes such as bites, tattoos and piercings. For HCV infections, injecting drug use was included as a category and non-occupational routes of transmission included sexual behaviours, household contacts, and other routes such as bites, tattoos and piercings.

For laboratory variables, ALT levels were recorded for those cases reported as being above the ULN. For HBV cases, HBV DNA levels, HBsAg, HBeAg, hepatitis Delta (HDV) and anti-HBc IgM were reported. For HCV, HCV RNA levels, anti-HCV, HCVc Ag and genotype were reported.

For clinical history at presentation, date of first diagnosis, time since acquisition of infection, reported fibrosis stage, cirrhosis or HCC and tests employed to determine fibrosis were collected. Late presentation was defined as either severe or advanced fibrosis (stage F3 or F4), cirrhosis or HCC [20].

We collected data on treatment start and, where applicable, end dates, treatment regimens, reported treatment outcomes of viral suppression for HBV and sustained virological response (SVR) for HCV were collected. Additional variables collected for HCV included previous treatment and reasons for repeat treatment.

### Statistical analysis

We performed descriptive analyses of individuals reported to have either HBV or HCV chronic infections, their treatment eligibility and treatment outcomes using STATA statistical software version 16.1 (Stata Corporation, Texas, United States).

## Results

The seven sentinel sites in the three countries were either infectious disease (four) or gastroenterology (three) tertiary clinics. Four clinics had a national, three a regional and one a district catchment population and the size of these ranged from 36,000 to 4,100,000 (Table 1). The seven sites accepted referrals from all healthcare services including low threshold and primary healthcare clinics. In Croatia and Spain, treatment and care costs are funded by national insurance. In Romania, treatment is financed by national insurance but some care and diagnostics costs are paid by either pharmaceutical companies or the individual patient.

The seven sentinel sites reported a total of 229 individuals with chronic HBV and 240 with chronic HCV infections. The University Emergency Hospital, Bucharest reported most individuals with chronic HBV infection (141, 62%) and the Hospital Universitario Valle Hebrón, Barcelona, reported the most individuals with chronic HCV infection (53, 22%) (Table 1).

### Chronic HBV infection

The majority of reported chronic HBV cases were male (56%, 127/228), and median age was 55 years (n = 226, interquartile range (IQR): 41–64). The two most commonly reported transmission categories for chronic HBV cases were non-occupational (25%, 29/114) and healthcare-associated infections (22%, 25/114) (Table 2). Due to the small number of cases, the two individuals who had acquired their infection through injecting drug use were included with other non-occupational transmission routes.

**Table 2:** Sociodemographic and clinical characteristics of chronic hepatitis B cases by country, hepatitis pilot sentinel surveillance system, Croatia, Romania, Spain, January–June 2019

Variables	Croatia		Romania		Spain		Total	
	Number of cases (total n = 24)	%	Number of cases (total n = 150)	%	Number of cases (total n = 55)	%	Number of cases (total n = 229)	%
**Socio-demographic variables**								
**Sex**	**24**	NA	**149**	NA	**55**	NA	**228**	NA
Male	15	63	78	52	34	62	127	56
Female	9	38	71	48	21	38	101	44
**Age**	**24**	NA	**147**	NA	**55**	NA	**226**	NA
Median age in years (IQR)	54 (47–62		60 (49–69)		35 (28–50)		55 (41–64)	
**Country of birth**	**12**	NA	**111**	NA	**55**	NA	**178**	NA
Same as reporting country	12	100	111	100	12	22	135	76
Other	0	0	0	0	43	78	43	24
**Transmission route**	**11**	NA	**90**	NA	**13**	NA	**114**	NA
Sexual	2	18	15	17	3	23	20	18
Mother-to-child	2	18	0	0	10	77	12	11
Any non-occupational^a^	5	45	24	27	0	0	29	25
Healthcare associated^b^	2	18	23	26	0	0	25	22
Any occupational^c^	0	0	16	18	0	0	16	14
Other	0	0	12	13	0	0	12	11
**Laboratory variables**								
**HCV co-infection**	**24**	NA	**150**	NA	**55**	NA	**229**	NA
HCV positive	3	13	6	4	0	0	9	4
**HDV coinfection**	**1**	NA	**86**	NA	**49**	NA	**136**	NA
HBV Delta positive	0	0	8	9	2	4	10	7
**HBV DNA levels**	**24**	NA	**43**	NA	**55**	NA	**122**	NA
Detected	22	92	18	42	51	93	91	75
**HBV e antigen**	**24**	NA	**88**	NA	**55**	NA	**180**	NA
HBeAg positive	2	8	20	23	1	2	29	16
**Alanine amino transferase**	**24**	NA	**148**	NA	**55**	NA	**227**	NA
Above upper limit normal	18	75	45	30	13	24	76	33
**Clinical variables**								
**Fibrosis stage**	**24**	NA	**42**	NA	**54**	NA	**120**	NA
F0 (no fibrosis)	1	4	17	41	39	72	57	48
F1 (minimal)	7	29	10	24	11	21	28	23
F2 (significant)	8	33	4	10	4	7	16	13
F3 (severe)	1	4	4	10	0	0	5	4
F4 (cirrhosis/advanced)	7	29	7	17	0	0	14	12
**Cirrhosis**	**24**	NA	**145**	NA	**55**	NA	**224**	NA
Diagnosed	2	8	15	10	0	0	17	8
**Hepatocellular carcinoma**	**24**	NA	**149**	NA	**55**	NA	**228**	NA
Diagnosed	2	8	5	3	0	0	7	3
**Late presentation** ^d^	**24**	NA	**42**	NA	**54**	NA	**120**	NA
Yes	8	33	12	29	0	0	20	17

HBeAg: hepatitis B e antigen; HBV: hepatitis B virus; HVC: hepatitis C virus; IQR: interquartile range; NA: not applicable.

Numbers in bold indicate total cases with complete information for each variable.

^a^Household contact, injecting drug use and other routes such as bites, tattoos and piercings.

^b^Transmission through blood and blood products, organs and tissues, haemodialysis as well as other exposure through hospitals, nursing homes, psychiatric institutions and dental services. This category refers mainly to patients exposed through healthcare settings, distinct from occupational exposure, which refers to staff.

^c^Includes needle stick injuries among healthcare workers.

^d^Late presentation defined as diagnosis of either fibrosis stage F3 or F4, cirrhosis or hepatocellular carcinoma.

Nine of 229 (4%) individuals with HBV had an HCV coinfection, of whom six were reported from sites in Romania, and 10 (7%) were coinfected with hepatitis D virus (HDV). Hepatitis B DNA was detected in 75% (91/122) of all the chronic HBV infections that had that DNA data available. The proportion of patients with HBV DNA detected was much lower in Romanian sites (42%, 18/43) than those in Croatia and Spain. Elevated ALT levels were reported in a third of chronic HBV infections (33%, 76/227), and 29% (35/120) of patients were diagnosed with F2 (significant fibrosis) stage or higher. Eight percent of patients (17/224) were diagnosed with cirrhosis and 3% (7/228) were diagnosed with hepatocellular carcinoma. Overall, late presentation of chronic HBV infection was reported for 17% (20/120) of cases (Table 2).

**Figure 1:**
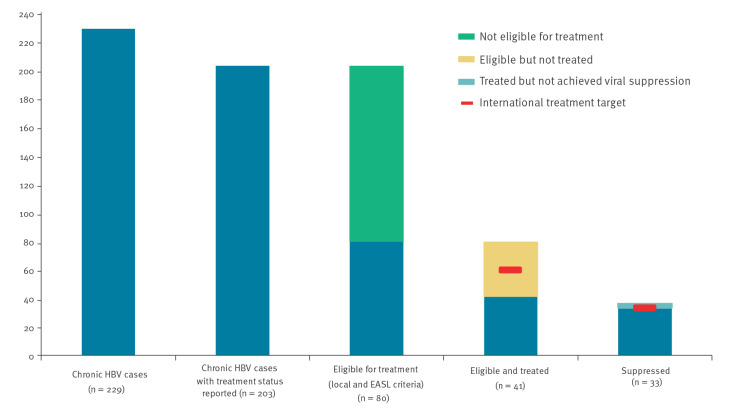
Continuum of care for chronic hepatitis B cases as reported in the hepatitis pilot sentinel surveillance system, Croatia, Romania, Spain, January–June 2019

EASL: European Association for the Study of the Liver; HBV: hepatitis B virus.

In the continuum of care, of the 229 individuals with chronic HBV infection, treatment status was reported for 203, of whom 80 (39%) fulfilled either the EASL or local treatment eligibility criteria (Figure 1). The WHO targets for > 75% of eligible individuals treated and ≥ 90% viral suppression in treated cases were generally missed as, of the 80 individuals eligible for treatment, only 51% (41/80) were reported as treated and 89% (33/37) of those treated had achieved viral suppression. Twenty-eight individuals with chronic HBV infection met local clinical eligibility criteria but were not reported as fulfilling the EASL eligibility criteria. Of these 28, 11 had received treatment.

While all reported individuals with an HBV infection were eligible for treatment at the sites in Croatia, only a minority were eligible at the sites in Romania (31%, 46/150) and Spain (18%, 10/55). Nearly all eligible chronic HBV cases at the sites in Croatia were treated (96%, 23/24), while 60% (6/10) were treated at the site in Spain and 26% (12/46) were treated at the sites in Romania (Table 3). Similar numbers of patients were treated with tenofovir (45%, 18/40) and lamivudine (48%, 19/40), with a minority treated with other drug combinations (8%, 3/40). Variations in the treatment regimen employed were reported. All patients were treated with tenofovir in Spain, 55% in Croatia (12/22) and none in Romania. The WHO target of ≥ 90% viral suppression in treated cases was met at the sites in Romania (100%, 11/11) but not those in Croatia (87%, 20/23) or Spain (67%, 2/3), although numbers at all sites were low. The median delay between diagnosis and treatment was 188 days (n = 33, IQR: 7–278 days) and between first attendance and treatment was 52 days (n = 49, IQR: 0–186 days) (Table 3).

**Table 3:** Eligibility and treatment of chronic hepatitis B cases with reported treatment status by country, hepatitis pilot sentinel surveillance system, Croatia, Romania, Spain, January–June 2019.

Reported treatment status	Croatia		Romania		Spain		Total	
	Number of cases (total n = 24)	%	Number of cases (total n = 150)	%	Number of cases (total n = 55)	%	Number of cases (total n = 203)	%
**Eligible for treatment**	**24**	**100**	**46**	**31**	**10**	**18**	**80**	**39**
**Eligibility treatment criteria**	**24**	NA	**46**	NA	**10**	NA	**80**	NA
EASL	18	75	24	52	10	100	52	73
Local	6	25	22	48	0	0	28	27
**Reported treatment status**	**24**	NA	**46**	NA	**10**	NA	**80**	NA
Treated	23	96	12	26	6	60	41	51
**Treatment regimen**	**22**	NA	**12**	NA	**6**	NA	**40**	NA
Lamivudine	7	32	12	100	0	0	19	48
Tenofovir	12	55	0	0	6	100	18	45
Other/multiple regimens	3	14	0	0	0	0	3	8
**Viral suppression**	**23**	NA	**11**	NA	**3**	NA	**37**	NA
Yes	20	87	11	100	2	67	33	89
**Diagnosis to treatment start**	**17**	NA	**12**	NA	**4**	NA	**33**	NA
Median days (IQR)	1,375 (94–3,495)		75 (7–189)		278 (187–301)		188 (87–1,375)	
**Attendance to treatment start**	**22**	NA	**12**	NA	**6**	NA	**40**	NA
Median days (IQR)	51 (0–92)		188 (48–192)		151 (14–259)		75 (18–189)	

EASL: European Association for the Study of the Liver; IQR: interquartile range; NA: not applicable.

Of the 229 participants in the study, treatment status was available for 203.

Numbers in bold indicate total cases with complete information for each variable.

Of the 52 individuals with chronic HBV eligible for treatment according to the EASL criteria, 63% (33/52) fulfilled the criterion for chronic hepatitis. Fulfilment of other remaining criteria ranged from 33% (17/52) diagnosed with cirrhosis to 4% (2/52) who were HBeAg-positive (Table 4). Overall, 58% (30/52) of chronic HBV cases that fulfilled the EASL criteria for treatment were treated, and percentage treated ranged from 18% of those diagnosed with cirrhosis (3/17) to 100% (2/2) for those who were HBeAg positive (Table 4).

**Table 4:** Distribution of individual eligibility criteria and proportions treated among 52 chronic hepatitis B cases meeting at least one treatment eligibility criteria^a^ recommended by the European Association for the Study of the Liver, hepatitis pilot sentinel surveillance system, Croatia, Romania, Spain, January–June 2019

Individual treatment criteria	Eligible for HBV treatment	Treated for HBV	
	n	n	%
**Diagnosed cirrhosis**	17	3	18
**Chronic hepatitis** ^b^	33	26	79
**Very high HBV DNA and ALT levels** ^c^	7	6	86
**HBeAg positivity** ^d^	2	2	100
**Other reasons** ^e^	9	6	67
**Total meeting at least one treatment eligibility criteria**	**52**	**30**	**58**

ALT: Alanine aminotransferase; HBeAg: hepatitis B e antigen; HBV: hepatitis B virus.

^a^ Clinical eligibility criteria as outlined in European guidelines for the management of hepatitis B virus infection (European Association for the Study of the Liver (EASL) 2017).

^b^ Clinical eligibility defined as HBV DNA > 2,000 IU/ml, ALT > ULN (40IU/ml) and/or at least moderate fibrosis (F2 or greater).

^c^ Clinical eligibility defined as HBV DNA > 20,000 IU/ml and ALT > 2xULN (80 IU/ml).

^d^ Clinical eligibility defined as HBVeAg-positive, normal ALT (< 40IU/ml), high (> 2,000 IU/ml) HBV DNA and aged 30 years or older.

^e^ Other treatment criteria including prevention of mother-to-child transmission or reactivation due to immunosuppression or chemotherapy.

### Chronic HCV infection

The majority of reported chronic HCV cases were female (53%, 127/240) and median age was 54 years (n = 239, IQR: 44–63) (Table 5). Injecting drug use was the most commonly reported route of transmission for HCV chronic infections (49%, 75/153), followed by healthcare-associated transmission (30%, 46/153). Data on genotype were reported for 138 HCV cases, of which 48 (35%) were genotype 1a, 36 (26%) genotype 1b, 41 (30%) were genotype 3 and the remainder (9%, 13/138) were genotypes 2 or 4. ALT levels above ULN were reported in 68% (162/240) of chronic HCV cases and 55% (130/238) were diagnosed with F2 (significant) stage of fibrosis or higher (Table 5). Twelve (5%, 12/237) reported cases were diagnosed with cirrhosis and seven (3%, 7/236) with hepatocellular carcinoma. Overall, over a third of chronic HCV cases (36%, 79/221) presented with evidence of late disease.

**Table 5:** Sociodemographic and clinical characteristics of chronic hepatitis C cases by country, hepatitis pilot sentinel surveillance system, Croatia, Romania, Spain, January–June 2019

Variables	Croatia		Romania		Spain		Total	
	Number of cases (total n = 92)	%	Number of cases (total n = 95)	%	Number of cases (total n = 53)	%	Number of cases (total n = 240)	%
**Sociodemographic variables**								
**Sex**	**92**	NA	**95**	NA	**53**	NA	**240**	NA
Male	59	64	21	22	33	62	113	47
Female	33	36	74	78	20	38	127	53
**Age**	**92**	NA	**95**	NA	**53**	NA	**240**	NA
Median age in years (IQR)	47 (41–55)		61 (52–68)		53 (43–62)		54 (44–63)	
**Country of birth**	**50**	NA	**94**	NA	**47**	NA	**191**	NA
Same as reporting country	50	100	93	99	36	77	179	94
Other	0	0	1	1	11	23	12	6
**Transmission route**	**75**	NA	**47**	NA	**31**	NA	**153**	NA
Non-occupational^a^	3	4	13	28	0	0	16	11
Injecting drug use	51	68	0	0	24	77	75	49
Healthcare associated^b^	19	25	20	43	7	23	46	30
Any occupational^c^	1	1	5	11	0	0	6	4
Other	1	1	9	19	0	0	10	7
**Laboratory variables**								
**HCV RNA**	**91**	NA	**94**	NA	**53**	NA	**238**	NA
Detected	91	100	94	100	53	100	238	100
**HCV core antigen**	**32**	NA	**3**	NA	**0**	NA	**35**	NA
Detected	31	97	2	67	0	0	33	94
**HCV genotype**	**89**	NA	**4**	NA	**45**	NA	**138**	NA
1a	39	43	1	25	8	18	48	35
1b	14	16	3	75	19	42	36	26
3	29	33	0	0	12	27	41	30
Other (genotypes 2 and 4)	7	8	0	0	6	13	13	9
**Alanine aminotransferase**	**93**	NA	**95**	NA	**52**	NA	**240**	NA
Above Upper Limit Normal	82	88	52	55	28	54	162	68
**Clinical variables**								
**Fibrosis stage**	**91**	NA	**94**	NA	**53**	NA	**238**	NA
F0 (no fibrosis)	11	12	17	18	21	40	49	21
F1 (minimal)	23	25	32	34	4	8	59	25
F2 (significant)	12	13	23	24	14	26	49	21
F3 (severe)	10	11	11	12	5	9	26	11
F4 (cirrhosis/advanced)	35	38	11	12	9	17	55	23
**Cirrhosis**	**91**	NA	**93**	NA	**53**	NA	**237**	NA
Diagnosed	7	8	5	5	0	0	12	5
**Hepatocellular carcinoma**	**91**	NA	**92**	NA	**53**	NA	**236**	NA
Diagnosed	5	5	1	1	1	2	7	3
**Late presentation** ^d^	**88**	NA	**81**	NA	**52**	NA	**221**	NA
Yes	44	50	20	25	15	29	79	36

HCV: hepatitis C virus; IQR: interquartile range; NA: not applicable.

Numbers in bold indicate total cases with complete information for each variable.

^a^ Sexual behaviours, household contacts, and other routes such as bites, tattoos and piercings.

^b^ Transmission through blood and blood products, organs and tissues, haemodialysis as well as other exposure through hospitals, nursing homes, psychiatric institutions and dental services. This category refers mainly to patients exposed through healthcare settings, distinct from occupational exposure, which refers to staff.

^c^ Includes needle stick injuries among healthcare workers.

^d^ Late presentation defined as diagnosis of either fibrosis stage F3 or F4, cirrhosis or hepatocellular carcinoma.

**Figure 2:**
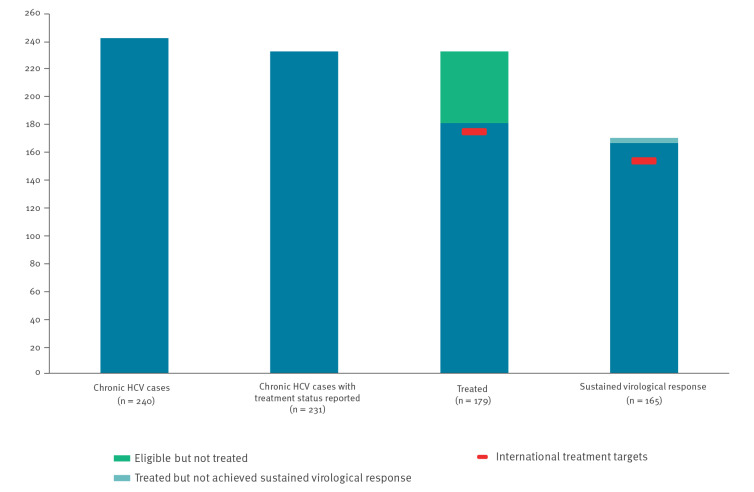
Continuum of care for chronic hepatitis C cases as reported by the hepatitis pilot sentinel surveillance system, Croatia, Romania, Spain, January–June 2019

Of the 240 individuals reported to have a chronic HCV infection, treatment status was available for 231 (Figure 2). For those 231 individuals, the WHO treatment target of at least 75% of eligible cases treated was achieved (77%, 179/231). Among the 169 cases for whom SVR was reported, the WHO target of 90% of treated cases achieving viral suppression was also met (98%, 165/169). Nearly all cases had ended their treatment by the end of the pilot period (99%, 177/178).

HCV: hepatitis C virus.

The proportion of individuals with chronic HCV being treated varied by country, with 94% of cases being treated at the Spanish site and less than three quarters of cases being treated at the sites in Croatia and Romania (70% and 74%, respectively). However, in all sites nearly all (>90%) of treated patients were reported as having achieved SVR. The median delay between diagnosis and treatment start was 356 days (n = 122, IQR: 95–3,576 days), with a median delay of 35 days (n = 178, IQR: 16–113 days) between first attendance and treatment initiation. Data on treatment regimen was reported for 176 cases, of which nearly all (99%, 174/176) were treated with direct acting antivirals for a median of 84 days (n = 161, IQR: 73–89 days) (Table 6).

**Table 6:** Treatment of chronic hepatitis C cases with reported treatment status, hepatitis pilot sentinel surveillance system, Croatia, Romania, Spain, January–June 2019

Reported treatment status	Croatia		Romania		Spain		Total	
	Number of cases (total n = 92)	%	Number of cases (total n = 95)	%	Number of cases (total n = 53)	%	Number of cases (total n = 231)	%
**Reported treatment status**	**88**	NA	**90**	NA	**53**	NA	**231**	NA
Treated	62	70	67	74	50	94	179	77
**Treatment regimen**	**59**	NA	**67**	NA	**50**	NA	**176**	NA
DAA regimen	59	100	65	97	50	100	174	99
Interferon regimen	0	0	2	3	0	0	2	1
Other/multiple	0	0	0	0	0	0	0	0
**Sustained viral response**	**59**	NA	**67**	NA	**43**	NA	**169**	NA
Achieved	58	98	67	100	40	93	165	98
**Ended treatment**	**61**	NA	**67**	NA	**49**	NA	**178**	NA
Yes	60	98	67	100	49	100	177	99
**Diagnosis to treatment start**	**52**	NA	**65**	NA	**5**	NA	**122**	NA
Median days (IQR)	522 (203–6,394)		296 (35–2,061)		9,932 (115–10,678)		356 (95–3,576)	
**Attendance to treatment start**	**62**	NA	**67**	NA	**49**	NA	**178**	NA
Median days (IQR)	152 (94–215)		21 (9–35)		26 (0–42)		35 (16–113)	
**Duration of treatment**	**59**	NA	**67**	NA	**35**	NA	**161**	NA
Median days (IQR)	85 (59–87)		83 (82–89)		84 (66–91)		84 (73–89)	

DAA: direct acting antiviral; IQR: interquartile range; NA: not applicable.

Numbers in bold indicate total cases with complete information for each variable.

Of the 240 participants in the study, treatment status was available for 231.

## Discussion

This pilot of a sentinel surveillance system for HBV and HCV provided data from clinical sites to monitor progress towards international treatment targets for hepatitis elimination. We report that the achievement of WHO treatment targets varied between HBV and HCV chronic infections and by country.

Overall, a minority (39%) of chronic HBV cases were reported as fulfilling either EASL or local treatment eligibility criteria, but just over half of those considered eligible for treatment were reported as being treated, thus missing the 2020 target of 75% of eligible HBV cases being treated [7]. The poor uptake of treatment for HBV has been identified by other investigators [21,22] and highlights the need to identify interventions to improve engagement and retention with treatment services [23]. Nonetheless, the low uptake rates of HBV treatment may also be a consequence of the of a long delay (median of 188 days) from first attendance to treatment initiation, which may be due to the practice of a lengthy evaluation of HBV chronic cases before treatment initiation [24]. Thus, the follow up period used in this study may not have been long enough to capture the initiation of treatment for all cases.

Reported treatment rates for HBV varied substantially between countries and may be due to the different types of clinics participating as much as issues of access, although in all three pilot countries the costs of HBV treatment are fully reimbursed. Nucleos(t)ide analogues with high barriers to resistance are the preferred treatment for chronic HBV [18,25], yet lamivudine therapy was reported for nearly half of treated HBV cases. The majority (89%) of HBV treated patients were reported as having achieved viral suppression, not quite achieving the target of 90% viral suppression as outlined in the European Action Plan [7], although this may be also due to an insufficient follow-up period.

Overall, the majority of patients with chronic HCV infections (77%) had been treated achieving the target of 75% of eligible patients having initiated treatment. Furthermore, over 90% of individuals treated had completed treatment and achieved SVR in line with European targets [7]. While the site in Spain had achieved the international target of 75% treated, Croatia and Romania had missed these by small margins (< 5%). The lower treatment rates in Croatia and Romania may be due to differences in clinical practice in the participating sites or differences between countries in the eligibility criteria or financial reimbursement of treatment costs. For example, international clinical guidelines state that all patients with recently acquired or chronic HCV infection should be treated without delay, including people who inject drugs (PWID) [17]. However, in both Croatia and Romania limited access to treatment by PWID was reported at the time of the study [26].

A recent survey of EU/EEA countries reported that only a minority (7/31) of countries were able to provide estimates of the number of individuals being treated for HBV. The same survey showed that although the majority (18/31) of countries were able to report the number of people being treated for HCV, only a minority (12/31) were able to provide data on HCV treatment outcomes [15]. Monitoring treatment and treatment outcomes is performed by a variety of means in different European countries including treatment cohorts [27,28]. However, the treatment cohorts are often established by research projects which recruit specific populations, close to further recruitment after the data collection period and may not provide national coverage as they are limited to specific areas or groups [29]. Data may also come from analysis of laboratory [30] and health insurance [31] data, but these often record limited information [32]. Our approach of enhanced sentinel reporting has been employed elsewhere [33] and has many advantages, including the relative ease and flexibility of data collection.

A major limitation of this pilot sentinel surveillance project was that treatment uptake was measured in only a small number of sites which did not encompass all the types of healthcare facilities offering treatment, such as primary care or low threshold clinics, nor were geographically representative [34]. Thus, the study population attending selected clinical centres may not be representative of patients in care nationally. Furthermore, data collected from a small number of sentinel sites may be biased by the results of single site and this may have occurred in this pilot as one site in Romania reported over 50% of all cases reported in the study. However, we observed similar levels of treatment uptake and viral suppression in this study as reported nationally for HBV in Romania and for all three pilot countries for HCV [15]. To improve representativeness in future studies, the sampling frame needs to include all types of clinical sites, from tertiary to low-threshold services, to encompass the different clinical pathways from diagnosis to treatment and to include a larger number of sites from different parts of participating countries. Achieving a fully representative sample may be challenging and so the option of obtaining good quality data from a few clinical sites together with an understanding of how the data could be biased may be the preferred option in some circumstances.

Furthermore, as we collected data on patients in care, we are not able to estimate the proportion eligible for treatment and treated for HBV or treated for HCV among all those diagnosed as some have not been linked to care. There are many reasons why people are not linked to care including not being referred, problems with access or cost and the ability to keep appointments, for example among those who are homeless or inject drugs [26,35]. The WHO target for linkage to care states that 90% of patients diagnosed with chronic HBV or HCV infections should be linked to care and adequately monitored [7]. In the most recent ECDC report on the monitoring of responses to the hepatitis B and C epidemics in EU/EEA countries [15], only three countries submitted enough data to be able to estimate the proportion of people diagnosed with hepatitis B and linked to care. In addition, no country submitted adequate data to allow this estimation for hepatitis C. This illustrated the challenges of being able to estimate the extent to whether the proportion of treated patients among those in care are representative of all those diagnosed. A further limitation is that data were collected for a period of up to one year which may raise questions on how representative these data are since patients with HBV are often evaluated for longer periods before treatment initiation, thus the duration of the treatment regimen itself may have been missed. We believe that there was minimal or no ascertainment bias by our inclusion of HBV and HCV cases that had ‘no report’ of either anti-HBc IgM or anti-HCV as having a chronic infection.

### Conclusion

Data collected through this pilot sentinel surveillance showed that international treatment targets for hepatitis were generally missed for HBV but met for HCV. The wide variation in treatment uptake and outcomes across sites highlights the importance of assessing progress towards to treatment targets in a wide range of clinics. Nonetheless, the pilot sentinel surveillance for hepatitis demonstrated that the data collected provided insights into treatment of and treatment outcomes of patients diagnosed with HBV and HCV not currently obtained through existing routine data collection. The proposed sentinel surveillance system provides a robust foundation with which to monitor treatment and treatment outcomes in hepatitis patients, but future scale-up should ensure the inclusion of a larger number of sites representative of both geography and different treatment clinical pathways.
